# Influenza virus vaccination in pediatric nephrotic syndrome significantly reduces rate of relapse and influenza virus infection as assessed in a nationwide survey

**DOI:** 10.1038/s41598-021-02644-x

**Published:** 2021-12-02

**Authors:** Shingo Ishimori, Takashi Ando, Kaori Kikunaga, Chikako Terano, Mai Sato, Fumiyo Komaki, Riku Hamada, Yuko Hamasaki, Yoshinori Araki, Yoshimitsu Gotoh, Koichi Nakanishi, Hitoshi Nakazato, Takeshi Matsuyama, Kazumoto Iijima, Norishige Yoshikawa, Shuichi Ito, Masataka Honda, Kenji Ishikura

**Affiliations:** 1 Department of Pediatrics, Takatsuki General Hospital, Osaka, Japan; 2 Renal and Urological Surgery Department, International University of Health and Welfare Hospital, Tochigi, Japan; 3 Department of Nephrology, Tokyo Metropolitan Children’s Medical Center, Tokyo, Japan; 4 Division of Nephrology and Rheumatology, National Center for Child Health and Development, Tokyo, Japan; 5 Department of Pediatrics, Kawasaki Saiwai Clinic, Kanagawa, Japan; 6 Department of Nephrology, Faculty of Medicine, Toho University, Tokyo, Japan; 7 Department of Pediatrics, National Hospital Organization Hokkaido Medical Center, Sapporo, Japan; 8 Department of Pediatric Nephrology, Japanese Red Cross Nagoya Daini Hospital, Nagoya, Japan; 9 Department of Child Health and Welfare (Pediatrics), Graduate School of Medicine, University of the Ryukyus, Okinawa, Japan; 10 Department of Pediatrics, Faculty of Life Sciences, Kumamoto University, Kumamoto, Japan; 11 Department of Pediatrics, Fussa Hospital, Tokyo, Japan; 12 Department of Pediatrics, Kobe University Graduate School of Medicine, Hyogo, Japan; 13 Clinical Research Center, Takatsuki General Hospital, Osaka, Japan; 14 Department of Pediatrics, Graduate School of Medicine, Yokohama City University, Yokosuka, Japan; 15 Department of Pediatrics, Kitasato University School of Medicine, 1-15-1 Kitazato, Minami-ku, Sagamihara city, Kanagawa, 2520374 Japan

**Keywords:** Paediatric kidney disease, Inactivated vaccines, Influenza virus

## Abstract

Although vaccination may precipitate relapses of nephrotic syndrome (NS) in children with idiopathic NS, no data are available regarding NS activity regarding influenza (flu) virus infections and NS relapses after receiving inactivated flu vaccines. We conducted a nationwide study of children aged 6 months to 15 years with idiopathic NS to assess the relationship between NS relapse, flu vaccination, and flu infections. We used a multivariate Poisson regression model (MPRM) to calculate the risk ratio (RR) for flu infection and for NS relapse in children with and without flu vaccination. Data of 306 children were assessed. The MPRM in all 306 children showed a significantly lower RR for flu infection (RR: 0.21, 95% confidence interval CI 0.11–0.38) and for NS relapse (RR: 0.22, 95% CI 0.14–0.35) in children receiving flu vaccination compared with unvaccinated children. In an additional MPRM only among 102 children receiving flu vaccination, they had a significantly lower risk for NS relapse during the post-vaccination period (RR: 0.31. 95% CI 017–0.56) compared with the pre-vaccination period. Although our study was observational, based on the favorable results of flu vaccinations regarding flu infections and NS relapse, the vaccine may be recommended for children with NS.

## Introduction

Idiopathic nephrotic syndrome (NS) requires long-term follow-up to manage relapse. The incidence of idiopathic NS is approximately 1 in 16 per 100,000 children^1^, over half of whom develop frequently relapsing NS (FRNS) or steroid-dependent NS (SDNS)^2^. To prevent NS relapse, these patients require prolonged steroid therapy and immunosuppressive agents^3,4^. These children have been the state of immunocompromised, in which they have the potential for recurrent or severe infections.

Children with chronic renal disease have a higher risk for severe influenza (flu) infections than healthy children^5–7^. Administering flu vaccines is effective in reducing the prevalence of flu infections and the risk of severe flu infection. Kidney disease: Improving Global Outcomes (KDIGO) guidelines suggest that children with NS should receive flu vaccination to reduce the risk of serious infections^8^. Pediatric patients with NS can be protected against flu infection using an inactivated influenza vaccine, even if they are being treated with glucocorticoids or other immunosuppressive drugs^5^. However, as an immunogenic stimulus, immunizations have been reported to trigger NS relapse in children^9–14^. Although inactivated flu vaccines could precipitate NS relapse^15,16^, available data regarding the relative risk of NS relapse related to flu vaccines have been scarce until recently. We previously evaluated NS relapse after 104 children with NS received an inactivated subunit-antigen influenza virus vaccine^17^. Compared with the NS relapse rate during the pre-vaccination period as a control, the rate within 1 month after receiving flu vaccination was slightly but non-significantly increased (1.19 vs. 1.23 times/person-year, risk ratio: 1.04, 95% confidence interval: 0.82–1.89, *p*=0.88). Limitations of our previous research included that it was a retrospective study in a single tertiary center and included no data of flu infections. Further studies using a larger sample at multiple centers were needed to investigate not only NS relapses but also flu infections after influenza vaccination.

Here, we report the findings of a nationwide cohort study among Japanese children with idiopathic NS in which we focus on the relationships among NS relapses, flu vaccines, and flu infections.

## Results

### Participant characteristics

**Figure 1:**
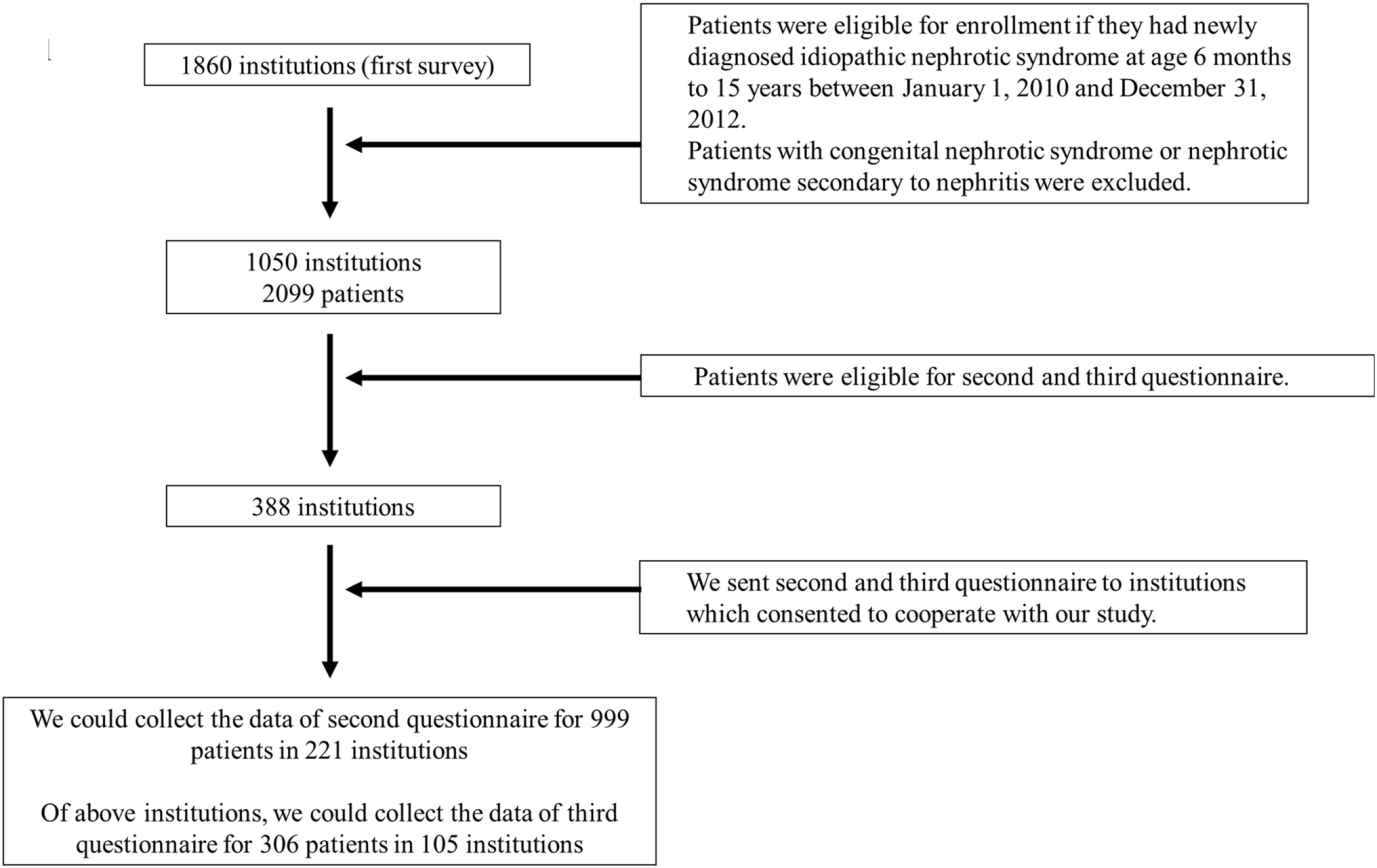
Flow chart of study design and populations. Of 388 institutions, 105 responded to the third questionnaire and data were collected for 306 children.

Overall, 105 of 388 institutions responded to the third questionnaire, and we collected data for 306 children. Figure 1 shows a flow chart of the study design and study populations.

We divided the children into two groups; those who received flu vaccination and those who did not receive flu vaccination. These children's clinical characteristics according to both the second and third questionnaires are shown in Table 1.

**Table 1:** Clinical characteristics of the patients.

	All (N = 306)	Flu vaccination (N = 102)	No flu vaccination (N = 204)	*p* value
** Background; second questionnaire**				
Age at onset of NS (years)	6.0 ± 4.1	5.6 ± 4.0	6.2 ± 4.2	0.26
Boy: Girl, *n* (%)	215: 91 (70.3%: 29.7%)	76: 26 (74.5%: 25.5%)	139: 65 (68.1%: 31.9%)	0.25
Gestational week*	38.7 ± 2.3	38.7 ± 2.2	38.7 ± 2.3	0.48
Birth weight ^†^	2945 ± 497	2983 ± 507	2925 ± 491	0.71
Family history of NS, *n* (%) ^‡^	9 (3.0%)	1 (1.0%)	8 (4.0%)	0.28
History of allergy, *n* (%) ^§^	114 (38.4%)	44 (43.6%)	70 (35.7%)	0.19
Asthma	36 (12.1%)	11 (10.9%)	25 (12.8%)	0.64
Atopic dermatitis	34 (11.4%)	12 (11.9%)	22 (11.2%)	0.87
Allergic rhinitis	56 (18.9%)	22 (21.8%)	34 (17.3%)	0.35
Allergic conjunctivitis	8 (2.7%)	4 (4.0%)	4 (2.0%)	0.45
Food allergy	28 (9.4%)	9 (8.9%)	19 (9.7%)	0.83
Other	6 (2.0%)	2 (2.0%)	4 (2.0%)	1.0
Hematuria at onset of NS, *n* (%) ^||^	95 (31.5%)	35 (35.0%)	60 (29.7%)	0.35
Past history of frequent-relapsing NS, *n* (%)	147 (48.0%)	39 (38.2%)	108 (52.9%)	0.015
Past history of steroid-dependent NS, *n* (%)	134 (43.8%)	38 (37.3%)	96 (47.1%)	0.1
Past history of steroid-resistant NS, *n* (%) ^¶^	29 (9.6%)	9 (8.9%)	20 (10.0%)	0.76
Renal biopsy, *n* (%) **	163 (54.3%)	50 (50.0%)	113 (55.6%)	0.45
Classification of renal histology				0.25
Minimal change	132 (81.0%)	40 (80.0%)	92 (81.4%)	
Focal segmental glomerular sclerosis	14 (8.6%)	7 (14.0%)	7 (6.2%)	
Diffuse mesangial proliferation	9 (5.5%)	0 (0.0%)	9 (8.0%)	
Other	8 (4.9%)	3 (6.0%)	5 (4.4%)	

NS; nephrotic syndrome, Flu; influenza virus, FRNS; frequently relapsing NS, SDNS; steroid-dependent NS, SRNS; steroid-resistant NS.

* Evaluated 246 children with data for gestational weeks. † Evaluated 254 children with data of birth weight. ‡ Evaluated 299 children with data for family history of SRNS. § Evaluated 297 children with data for history of allergy. || Evaluated 302 children with data for hematuria at onset of NS. ¶ Evaluated 301 children with data of past history of SRNS. ** Evaluated 300 children with data of renal biopsy.

### Flu infection

**Figure 2:**
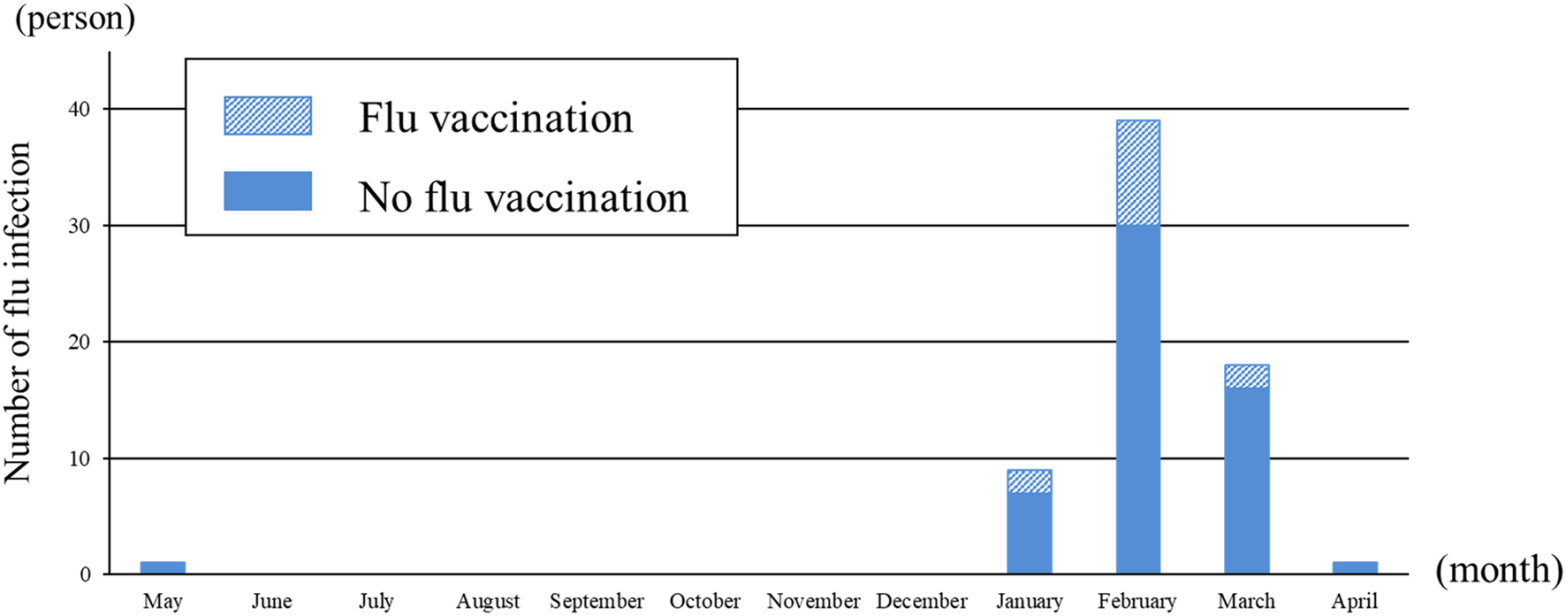
Number of influenza virus infections. Seasonal distribution of influenza (flu) infections was from January to May. February had the highest number of flu infections.

Table 2 shows that 65 children (21.2%) developed flu infection between 1 May 2015 and 31 April 2016. Compared with children who received flu vaccination, the flu infection rate among children without flu vaccination was significantly higher (12.7 vs. 25.4%, *p* = 0.01) . The diagnostic method of flu infections and the influenza strain were evaluated in 62 children (68 flu infection cases). All 13 infected children who received flu vaccination and 54 of 55 infected children who did not receive flu vaccination were diagnosed using rapid antigen detection tests and nasopharyngeal swab samples; 6 vaccinated and 25 unvaccinated children had influenza A infection, and 7 vaccinated and 29 unvaccinated children had influenza B infection. Only one unvaccinated patient was clinically diagnosed with flu infection. Figure 2 shows that the seasonal distribution of flu infections during the 2015/16 season was from January to May, and the highest number of flu infections was in February.

**Table 2:** Flu infections and nephrotic syndrome relapses.

	All (N = 306)	Flu vaccination (N = 102)	No flu vaccination (N = 204)	*p* value
Total number of flu infected patient, *n* (%)	65 (21.2%)	13 (12.7%)	52 (25.4%)	0.01
Total number of flu infection, *times* *	67	13	54	1.0
Total number of antigen A flu infection, *times* (%)	31 (46.3%)	6 (46.2%)	25 (46.3%)	
Total number of antigen B flu infection, *times* (%)	31 (53.7%)	7 (53.4%)	29 (53.7%)	
Number of children had NS relapse, *n* (%)	100 (32.7%)	30 (29.4%)	70 (34.3%)	0.39
Total number of NS relapse, *times* (times/person-year)	190 (0.62)	48 (0.25)	142 (0.74)	< 0.0001

NS; nephrotic syndrome, Flu; influenza virus, * Of 65 children with flu infection, 67 times diagnosed using rapid antigen detection tests with nasopharyngeal swab samples were evaluated.

### Results of multivariate analysis to evaluate the risk ratio for flu infection

Table 3 shows the results of multivariate analysis in the 306 children who received and did not receive flu vaccination.

**Table 3:** Risk ratio for flu infectionin 306 children who received and did not receive flu vaccination (multivariate analysis). (a) all influenza infections, (b) influenzaA infections, (c) influenzaB infections.

Variable	Risk ratio	95% Confidence interval	*P* value
** (a) For all flu infection**			
Received flu vaccines (reference: no vaccines)	0.21	0.11–0.38	< 0.0001
Female (reference: male)	1.49	0.92–2.42	0.10
Age at onset of NS	0.90	0.84–0.97	0.003
Past history of FRNS or SDNS or SRNS (reference: no history of FRNS or SDNS or SRNS)	0.77	0.47–1.23	0.27
On February (reference: other months)	16.58	10.23–26.89	< 0.0001
** (b) For flu A infection**			
Received flu vaccines (reference: no vaccines)	0.21	0.09–0.50	0.0004
Female (reference: male)	1.78	0.89–3.58	0.10
Age at onset of NS	0.91	0.83–1.00	0.053
Past history of FRNS or SDNS or SRNS (reference: no history of FRNS or SDNS or SRNS)	0.53	0.29–1.05	0.07
On February (reference: other months)	19.80	9.64–40.65	< 0.0001
** (c) For flu B infection**			
Received flu vaccines (reference: no vaccines)	0.20	0.09–0.46	0.0002
Female (reference: male)	1.27	0.65–2.50	0.48
Age at onset of NS	0.90	0.82–0.99	0.027
Past history of FRNS or SDNS or SRNS (reference: no history of FRNS or SDNS or SRNS)	1.08	0.54–2.14	0.83
On February (reference: other months)	14.27	7.40–27.50	< 0.0001

NS; nephrotic syndrome, Flu; influenza virus, FRNS; frequently relapsing NS, SDNS; steroid-dependent NS, SRNS; steroid-resistant NS.

The multivariate Poisson regression model (MPRM) showed statistical evidence of a higher risk ratio for all flu infections in February than in other months (risk ratio: 16.58. 95% confidence interval: 10.23–26.89), and a lower risk ratio for flu infections in children with older age at the onset of NS (risk ratio: 0.90. 95% confidence interval: 0.84–0.97) and in children who received flu vaccination (risk ratio: 0.21. 95% confidence interval: 0.11–0.38) (Table 3a). According to flu antigen, the data of risk ratio for influenza B infection were similar to those for all influenza strains (Table 3c); however, the age at onset of NS was not significantly related to influenza A infection (Table 3b). Flu infection in children with NS was not significantly related to sex or history of steroid-resistant NS (SRNS).

### NS relapse

**Figure 3:**
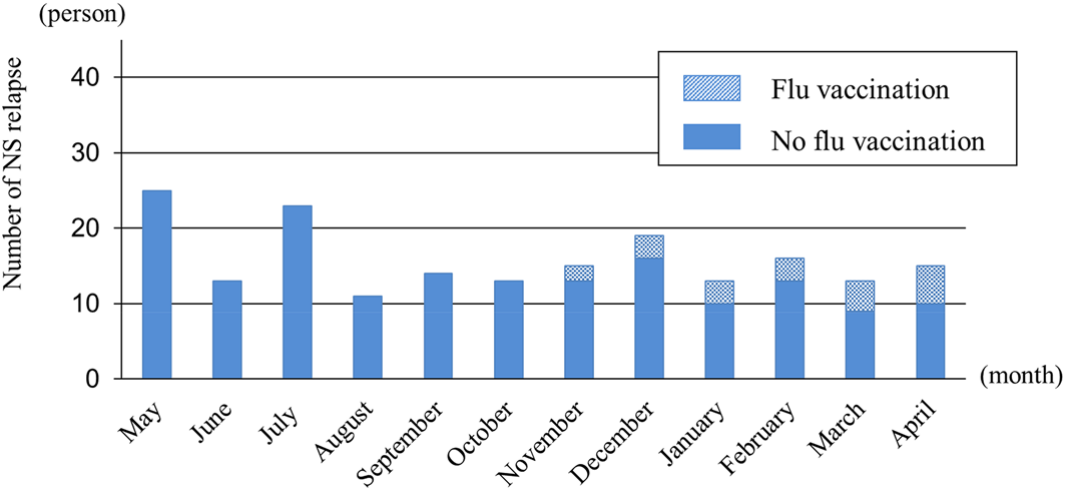
Number of nephrotic syndrome (NS) relapses. There was no seasonal distribution of NS relapse.

In total, 100 children (32.7%) had NS relapse between 1 May 2015 and 31 April 2016, and there were 190 total NS relapses (Table 2). The proportion of children with NS relapse was slightly higher in children who were not vaccinated compared with their vaccinated counterparts; however, this did not reach statistical significance. Furthermore, the number of NS relapses among children without the flu vaccination was significantly higher compared with children who received the flu vaccination (0.25 vs 0.74 times/person-year, *p* < 0.0001). There was no seasonal distribution of NS relapse (Fig. 3).

### Results of multivariate analysis to evaluate the risk ratio for NS relapse

In Table 4, the MPRM for 306 children that received and did not receive flu vaccination showed a significantly lower risk ratio for NS relapse in children who received flu vaccination (risk ratio: 0.22. 95% confidence interval: 0.14–0.35), and a higher risk ratio for NS relapse in children with a history of FRNS or SDNS or SRNS (risk ratio: 2.80. 95% confidence interval: 1.90–4.11). Sex and age at onset of NS were not independent risk factors for NS relapse in this multivariate analysis.

**Table 4:** Risk ratio for nephrotic syndrome relapse in 306 children who received and did not receive flu vaccination (multivariate analysis).

Variable	Risk ratio	95% Confidence interval	*P* value
** For NS relapse**			
Received flu vaccines (reference: no vaccines)	0.22	0.14–0.35	< 0.0001
Female (reference: male)	1.08	0.80–1.47	0.61
Age at onset of NS	0.99	0.95–1.02	0.41
Past history of FRNS or SDNS or SRNS (reference: no history of FRNS or SDNS or SRNS)	2.80	1.90–4.11	< 0.0001

NS; nephrotic syndrome, Flu; influenza virus, FRNS; frequently relapsing NS, SDNS; steroid-dependent NS, SRNS; steroid-resistant NS.

Table 5 shows the results of the MPRM conducted among the 102 children who received flu vaccination to evaluate the risk of NS relapse during the period after flu vaccination in comparison with before flu vaccination. Taking glucocorticoids at the time of flu vaccination was a risk factor significantly associated with NS relapse (risk ratio: 3.17. 95% confidence interval: 1.74–5.80). Compared with the pre-vaccination period, a significantly lower risk for NS relapse during the post-vaccination period (risk ratio: 0.31. 95% confidence interval: 017–0.56). There was no significant relationship between NS relapse and sex or being on cyclosporine or mizoribine at the time of flu vaccination.

**Table 5:** Risk ratio for nephrotic syndrome relapse among 102 children who received flu vaccination (multivariate analysis).

Variable	Risk ratio	95% Confidence interval	*P* value
** For NS relapse**			
Post vaccination period (reference: pre vaccination period)	0.31	0.17–0.56	< 0.0001
Female (reference: male)	0.81	0.36–1.86	0.62
The state of being on medicines at flu vaccinations			
Being on glucocorticoid (reference: no glucocorticoid)	3.17	1.74–5.80	0.0002
Being on Cyclosporine (reference: no Cyclosporine)	0.82	0.43–1.58	0.55
Being on Mizoribine (reference: no Mizoribine)	0.56	0.22–1.44	0.23

NS; nephrotic syndrome, Flu; influenza virus.

## Discussion

In the present study, we conducted a nationwide cohort study comprising 306 children with idiopathic NS, and showed that children who received flu vaccination had significantly fewer flu infections and NS relapses than those who did not receive flu vaccination. Additionally, the former group showed a significantly lower NS relapse risk during the post-vaccination period compared with the pre-vaccination period.

In this cohort, we examined the relationship among flu vaccination, flu infections, and NS relapses. Multivariate analysis revealed a significantly lower risk ratio for NS relapse in children who received flu vaccination. It has long been considered that vaccination itself can precipitate NS relapse as an immunogenic stimulation; however, the in-depth mechanism of immunogenic pathogenesis has not been elucidated. Several clinical studies have clinically suggested that the varicella vaccine^10,11^, meningococcal C conjugate vaccine^12^, and hepatitis B virus vaccine^13^ trigger NS relapses in children with NS. Nevertheless, that vaccination can precipitate NS relapse is not applicable to all vaccines. The 7-valent pneumococcal conjugate vaccine has not been found to be related to the risk of NS relapse in association with vaccination^14^. Focusing on flu vaccination in patients with NS, Fernandes et al. described a case report of NS relapse following the use of a monovalent whole-virion inactivated flu vaccine^15^. However, in our nationwide analysis investigating both vaccinated and unvaccinated patients at a number of institutions, we revealed a significant decrease in the NS relapse risk among children who received flu vaccination. Our results provide affirmative findings in comparison with our previous study targeting 104 vaccinated children with NS in a single facility. According to our past and present studies, it may be unnecessary to avoid flu vaccination in children with NS for fear of NS relapse.

In children with NS, an immunogenic stimulus related to infection or vaccination might trigger an NS relapse caused by the activation of T cells in an acute process^12,18^. However, no studies have reported that infections or vaccinations were associated with a decreased rate of NS relapse immunologically. We expect that the prevention of flu infection in NS children who received the flu vaccine might lead to a decrease in the rate of NS relapse regarding flu infection. Moreover, because flu vaccinations preventive flu infection as well as other infectious diseases^19,20^, flu vaccinations might also prevent NS relapse triggered by other infections.

This cohort study provided statistical evidence of a lower risk ratio for flu infection among children who receive flu vaccination. It has been reported that upper respiratory viral infections in pediatric patients induce NS relapse. McDonald et al. showed that respiratory viruses such as respiratory syncytial virus, parainfluenza virus, varicella zoster virus, and adenovirus are associated with NS exacerbation and relapse in children^21^. Similarly, NS relapse following influenza virus infection has been reported, including infection with the 2009 pandemic H1N1 virus^18,21^. Therefore, reducing flu infections in children with NS is desirable. The effectiveness of flu vaccination in pediatric patients with NS is proven serologically, with adequate antibody responses at 6 months post-vaccination^16^. In addition, our survey demonstrated the clinical efficacy of flu vaccination among children with NS in preventing flu infections.

We found that being on glucocorticoids when receiving flu vaccination was a risk factor significantly associated with NS relapse among children with NS. Pediatric patients treated with steroids at the time of flu vaccination would means on the state soon after the last NS relapse. Thus, NS relapses might be easy to trigger in children who are treated with steroids at the time of receiving flu vaccination owing to taking glucocorticoids being a marker of recent NS relapse.

The present study had several limitations. First, a significant difference in the history of FRNS between children with and without flu vaccination was a confounding factor. We could not adjust the data to control for this, even when making adjustments in the multivariate analysis, because this was an uncontrolled study. Each physician may have used the type of NS as a selection criterion when deciding to recommend flu vaccination to patients. Regardless, we evaluated many influencing factors for flu infection and NS relapse to adjust for confounding factors as much as possible. Nevertheless, we should recognize this confounding factor in the present analysis and therefore, the results regarding a lower risk for NS relapse in children who received the flu vaccination cannot be applied to all children with NS. We should interpret these confounding factors carefully, with the understanding that differences in their background often occur in this type of survey. Although a randomized trial might resolve these confounding factors, the methodology of the present study is easier to perform when establishing the side effects of vaccinations as a primary endpoint, as well as accumulating the required number of patients to detect differences. Moreover, we expect that there are other unrecognized confounding factors between children with and without flu vaccination. Second, we could not collect data of all events during the observation period. With further details, including data of whether fever, rhinorrhea, coughing, and vomiting resulting from both bacterial and viral infections triggered NS relapse, we might be able to conclude that flu vaccination could prevent NS relapse in terms of direct management of various infections. Third, we had no data regarding the precise interval between the timing of flu vaccination and NS relapses in each individual. Because the side effects of the inactivated subunit-antigen flu vaccine generally occur within 2–4 weeks of administration, NS relapses within 1 month after vaccination were possibly triggered by vaccination itself. However, differences in the state of NS activity during the pre- and post-vaccination periods are worth evaluating to determine whether there is a significantly lower risk for NS relapse in the post-vaccination period as compared with the pre-vaccination period. Fourth, flu infection was determined using only rapid antigen detection tests. This type of test is notoriously inaccurate, with a low sensitivity (approximately 60%) but high specificity (> 95%)^22^. However, Keitel et al. reported the sensitivity of rapid antigen detection tests increased to 92% when performed between 24 and 48 h after the onset of symptoms^23^. This study was conducted in Japan where it is common to perform rapid antigen detection tests between 24 and 48 h after the onset of symptoms. Although we should consider this bias of inaccuracy in our study, the concordance rate in our patients was not as inaccurate as those in the article by Chartland. Finally, vaccine policies in each center are not uniform. Some facilities have a policy of administering inactivated subunit-antigen flu vaccines to children with NS depending on their steroid dose, and other centers have different policies depending on the period from onset of NS or last NS relapse.

In conclusion, although our study was observational, based on the favorable results of flu vaccination against flu infection and NS relapse, the vaccine may be recommended for children with NS regardless of immunosuppressant use at the time of vaccination.

## Methods

### Study design and populations

The Japanese Pediatric Survey Holding Information of Nephrotic Syndrome (JP-SHINE) study, a nationwide cohort study, was established by the Japanese Study Group of Renal Disease in Children. The first survey was sent on 4 April 2014 to 1860 institutions throughout Japan, including all universities and children’s and general hospitals with more than 20 beds. Patients were eligible if they were newly diagnosed with idiopathic NS between 1 January 2010 and 31 December 2012, were between 6 months and 15 years old, and if they had been treated for up to 3 years during this time. Patients with congenital nephrotic syndrome or nephrotic syndrome secondary to nephritis were excluded. The first questionnaire, which was designed to record the number of children with idiopathic NS at each institution and their basic information, was sent to 1860 institutions. Of these, 1050 institutions (56.5%) responded to the first questionnaire^24^. We sent both questionnaires to 388 institutions that consented to cooperate with our study and which were eligible for a second and third questionnaire. The second questionnaire for patients who fulfilled the eligibility criteria was sent to 388 institutions and recorded information including patients’ basic characteristics, renal biopsy, idiopathic NS complications, steroid therapy side effects, and prognosis. We collected data for 999 patients^25^. In the third questionnaire, we recorded the age when the patient received the first inactivated subunit-antigen flu vaccine, the total number of flu vaccinations received, total number of flu infections, total number of NS relapses, rituximab (RTX) use at the first flu vaccination, and immunosuppressant use at the first flu vaccination (cyclosporine, mycophenolate mofetil, mizoribine, cyclophosphamide, tacrolimus) between 1 May 2015 and 31 April 2016.

### Definitions

In the present study, the definitions of general conditions in pediatric NS were according to clinical guidelines issued by the Japanese Society for Pediatric Nephrology^26,27^. Idiopathic NS in children was defined as hypoalbuminemia (serum albumin levels ≤ 2.5 g/dL) and severe proteinuria (≥ 40 mg/h/m^2^ in pooled nighttime urine or an early morning urine protein creatinine (Cr) ratio > 2.0 g/g Cr). Complete remission was defined as a urine protein creatinine ratio < 0.2 g/g Cr or ≤ negative protein on early morning urine dipstick testing for 3 consecutive days. SSNS was defined as complete remission in < 4 weeks after starting daily prednisolone therapy. NS relapse was defined as ≥ 3 + protein on early morning urine dipstick testing for 3 consecutive days. FRNS was defined as two or more relapses within 6 months after initial response or four or more relapses within any consecutive 12-month period. SDNS was defined as two consecutive relapses occurring during steroid therapy or within 2 weeks of treatment cessation. SRNS was defined as the absence of complete remission after a 4-week course of oral prednisolone 60 mg/m^2^/day. RTX therapy duration was defined as the period from the day of rituximab administration to the day of B-cell recovery (CD19 + B-cell count ≥ 1% of total lymphocytes).

Influenza virus infection was diagnosed using rapid antigen detection tests with a nasopharyngeal swab sample or was clinically diagnosed by an attending pediatrician. In Japan, children younger than 13 years old generally receive flu vaccination twice per year and those over 13 years old are vaccinated once per year. In children who received two vaccinations in the same year, we defined the “first flu vaccine” as their first vaccination of the two. Antigen strains in inactivated flu vaccines between 2015 and 2016 were A/California/7/2009 (X-179A) (H1N1) pdm09, A/Switzerland/9,715,293/2013 NIB-88 (H3N2), B/Phuket/3073/2013, and B/Texas/2/2013.

### Steroid therapy and immunosuppressants

We adopted the modified International Study of Kidney Diseases in Children protocol, as shown in the Japanese pediatric idiopathic NS guideline^3,27–29^, and 63% of children were treated with the protocol, as follows. The initial 8-week treatment protocol was 60 mg/m^2^/day prednisolone (maximum daily dosage 60 mg) for 4 weeks, followed by 40 mg/m^2^/day (maximum daily dosage 40 mg) on alternate days for 4 weeks^25^.Thirty percent of children were treated with the prolonged protocol, which was initial treatment using a prolonged protocol of 60 mg/m^2^/day prednisolone (maximum daily dosage 60 mg) for 4 weeks, followed by 40 mg/m^2^/day (maximum daily dosage 40 mg) on alternate days, tapered over 2–6 months. Treatment in the remaining 7% of children was unknown. Immunosuppressants used between 1 May 2015 and 31 April 2016 were cyclosporine in 55%, mizoribine in 39%, cyclophosphamide in 13%, mycophenolate mofetil in 11%, RTX in 11%, and tacrolimus in 2% of patients.

### Ethics

This study was conducted according to the principles of the Declaration of Helsinki and following the ethical guidelines for Medical and Health Research Involving Human Subjects of the Ministry of Health, Labour and Welfare in Japan. Yokohama City University Hospital's central ethics review board approved this study (approval number: 1509001). This ethics committee clearly stated that the researchers did not need to obtain informed consent, because complete data in this study were collected from patient medical records. In accordance with this statement, informed consent was not required from patients or their parents in this study. According to the guidelines and the institutional ethics review board for the patients’ benefit, the protocol was displayed publicly in a poster at each hospital, and each patient or their family had the opportunity to refuse to be included in this analysis.

### Statistical analysis

The calculated sample size was 200–300 children (100–150 per group). We set the ratio of the group those who received flu vaccination and those who did not receive flu vaccination as 1:1. We needed approximately 30 events (children with flu infection) in our analysis, because we assumed that the number of events was greater than 10–15 in the groups that did and did not receive the flu vaccination. The number of children aged under 15 years in Japan is 1,500 million. The estimated number of pediatric outpatients aged under 15 years was assumed to be 500 million cumulatively, based on pediatric sentinel sites. If the rate of flu infection diagnosis is assumed to be 40%, the estimated number of children diagnosed with flu infection is 200 million (13.3%: 200 million divided by 1,500 million). As a result, the sample size was set as 225 children (13.3%: 30 divided by 225 children), which was calculated to satisfy these assumptions. We used *t*-tests for continuous values and the chi-square and Fisher’s exact test for categorical values. All data were expressed as mean ± standard deviation or number (percentage). We used a MPRM for children who received or did not receive flu vaccination to calculate the risk ratio for flu infection, adjusted for receiving or not receiving flu vaccination, sex, age at NS onset, history of FRNS or SDNS or SRNS, flu infection in February or another month, and the risk ratio for NS relapse adjusted for whether the patients received flu vaccination, sex, age at NS onset, and history of FRNS or SDNS or SRNS. The classification of renal biopsy and history of NS type were correlated and not independent (for example, children with focal segmental glomerular sclerosis were likely to have FRNS/SDNS/SRNS). For this reason and because there was a significant difference in history of FRNS between the two groups, we chose history of NS types (the reason for unifying the three types of NS was that they were not independent of each other and could overlap in one patient).To evaluate the risk of NS relapse after flu vaccination, we used the MPRM only among children who received flu vaccination to calculate the risk ratio for NS relapse, adjusted for the post-vaccination period or pre-vaccination period, sex, and whether the patient was taking glucocorticoids or various immunosuppressants at flu vaccination. Taking medication at the time of vaccination is an important factor for vaccine studies. Moreover, history of NS was not added because this type correlated with medicine use at vaccination (cyclophosphamide, mycophenolate mofetil, rituximab, and tacrolimus were excluded because the rate of children using these medicines was low, making it difficult to estimate statistically). A *P-*value < 0.05 was considered statistically significant. We used SAS software package for Windows, release 9.4 (SAS Institute Inc. Cary, NC, USA) to perform all statistical analyses.

## Data Availability

Data are available upon reasonable request.
